# Accessibility, availability and affordability of anti-malarials in a rural district in Kenya after implementation of a national subsidy scheme

**DOI:** 10.1186/1475-2875-10-316

**Published:** 2011-10-26

**Authors:** Nathan Smith, Andrew Obala, Chrispinus Simiyu, Diana Menya, Barasa Khwa-Otsyula, Wendy Prudhomme O'Meara

**Affiliations:** 1 Duke Global Health Institute, Trent Hall, Durham, North Carolina, USA; 2 Moi University School of Medicine, Nandi Rd, Eldoret, Kenya; 3 Webuye Demographic Surveillance Site Scientific Steering Committee, Eldoret, Kenya; 4 Moi University School of Public Health, Nandi Rd, Eldoret, Kenya; 5 Duke University School of Medicine, Division of Infectious Diseases, Durham, North Carolina, USA

## Abstract

**Background:**

Poor access to prompt and effective treatment for malaria contributes to high mortality and severe morbidity. In Kenya, it is estimated that only 12% of children receive anti-malarials for their fever within 24 hours. The first point of care for many fevers is a local medicine retailer, such as a pharmacy or chemist. The role of the medicine retailer as an important distribution point for malaria medicines has been recognized and several different strategies have been used to improve the services that these retailers provide. Despite these efforts, many mothers still purchase ineffective drugs because they are less expensive than effective artemisinin combination therapy (ACT). One strategy that is being piloted in several countries is an international subsidy targeted at anti-malarials supplied through the retail sector. The goal of this strategy is to make ACT as affordable as ineffective alternatives. The programme, called the Affordable Medicines Facility - malaria was rolled out in Kenya in August 2010.

**Methods:**

In December 2010, the affordability and accessibility of malaria medicines in a rural district in Kenya were evaluated using a complete census of all public and private facilities, chemists, pharmacists, and other malaria medicine retailers within the Webuye Demographic Surveillance Area. Availability, types, and prices of anti-malarials were assessed. There are 13 public or mission facilities and 97 medicine retailers (registered and unregistered).

**Results:**

The average distance from a home to the nearest public health facility is 2 km, but the average distance to the nearest medicine retailer is half that. Quinine is the most frequently stocked anti-malarial (61% of retailers). More medicine retailers stocked sulphadoxine-pyramethamine (SP; 57%) than ACT (44%). Eleven percent of retailers stocked AMFm subsidized artemether-lumefantrine (AL). No retailers had chloroquine in stock and only five were selling artemisinin monotherapy. The mean price of any brand of AL, the recommended first-line drug in Kenya, was $2.7 USD. Brands purchased under the AMFm programme cost 40% less than non-AMFm brands. Artemisinin monotherapies cost on average more than twice as much as AMFm-brand AL. SP cost only $0.5, a fraction of the price of ACT.

**Conclusions:**

AMFm-subsidized anti-malarials are considerably less expensive than unsubsidized AL, but the price difference between effective and ineffective therapies is still large.

## Background

Malaria is one of the most important of the parasitic diseases of mankind, causing almost 5 billion clinical episodes in endemic countries annually, with more than 90% of this burden occurring in sub-Saharan Africa [1]. Malaria can be successfully treated with an appropriate course of anti-malarials. However, if treatment is delayed, malaria can quickly become life-threatening, particularly for children. Inappropriate treatment with ineffective drugs, especially drugs to which a high degree of resistance exists, can also lead to severe complications and death. For these reasons, prompt and effective treatment is a cornerstone of malaria control programmes.

In many countries, treatment for fever and malaria through self-medication with anti-malarials bought over-the-counter (OTC) from drug vendors is common [2-5]. This channel is increasingly being considered as a viable option for improving drug availability to malaria infected individuals, particularly those located further away from public health facilities [6-10]. For example, in rural Tanzania where the majority of people seek malaria treatment from retail drug sellers, the government established accredited drug dispensing outlet (ADDO) as a private sector supplement for the distribution of subsidized ACT in order to increase access to the first-line anti-malarial in rural and underserved areas [8].

However, accessing effective anti-malarials through these outlets remains problematic [2,6,11]. Several factors contribute to poor access, chief among them being cost, and distance to an outlet [12]. Chima *et al *[13] in analyzing the cost of malaria treatment in Africa found it ranged between US$0.41 and US$3.88 per person, which is beyond a household with a modest income. In Burundi, artemisinin combination therapies (ACT) in the private retail sector cost the equivalent of 1.5 days wages [14]. The situation is not any different in rural Kenya where malaria is endemic, and the cost of effective treatment remains prohibitive to the most vulnerable [15].

Recently, a global subsidy has been launched that aims to reduce the price of effective artemisinin combination therapy (ACT) supplied through the retail sector and make them more affordable than ineffective, older anti-malarials. This approach not only addresses the underlying economic considerations for the consumer, but also allows control of the quality of anti-malarials purchased by this programme. The subsidy, entitled Affordable Medicines Facility-malaria (AMFm) is a recent initiative by the international community intended to address ACT pricing and improve coverage. AMFm was launched in Kenya in August 2010.

Five months after the roll-out of AMFm in Kenya, a survey was undertaken of all public health facilities and malaria medicine retailers, including private clinics, chemists, pharmacies and other specialized drug stores, that serve residents of the Webuye Health and Demographic Surveillance Site (WHDSS) in western Kenya. The survey was designed to measure three dimensions of access to ACT; accessibility, availability, and affordability. The available anti-malarial drugs, reported stock-outs, types and brand names, and prices of anti-malarials in the WHDSS in western Kenya were measured following the roll-out of the AMFm subsidy. The average distance from a household to the nearest public health facility and the distance to the nearest medicine vendor were estimated to understand the reasons for choosing treatment through malaria medicine retailers.

## Methods

### Study area

The study was carried out in the Webuye Health and Demographic Surveillance Site (WHDSS). The WHDSS is located in Bungoma East District in Western Province, Kenya. The WHDSS provides longitudinal surveillance through twice-annual household surveys to a population of 70,000 people living in six administrative sublocations. Malaria transmission is year round with seasonal peaks following the rains in June. Transmission is moderate to high. EIR is estimated to be 29 infectious bites per person per year and parasite prevalence is 55% in asymptomatic children during the rainy season [16]. Most families engage in subsistence farming and animal husbandry. There are large sugar cane plantations and processing plants that employ seasonal labour from the community.

### Census of facilities

All the major roads in the study area were mapped using a Garmin E-trex handheld GPS unit. Major town centres were marked. A team of 10 fieldworkers was trained to use the GPS units and each was sent out on a motorbike to cover a specific area. The field worker started in a designated market centre and identified all potential medicine retailers and private health facilities. They took the name and GPS coordinates of each one. They then asked the shop owner if there were any other places to buy medicines. They visited the next medicine retailer and continued inquiring about other medicine retailers until no new retailers were identified. In order to identify all retailers that might be accessed by those living within the DSS, all medicine retailers and clinics within 5 km of the border of the DSS were included, except on the north-eastern border, where a river marks the boundary of the DSS. A complete listing of public health facilities was obtained from the District Health Management Team. Each one was visited and the coordinates were recorded.

### Survey

Field workers were trained to administer the survey tool and a pilot study was conducted outside of the study area. Field workers visited each medicine retailer mapped during the census. Shops that sold general goods including a small selection of painkillers or other medicines were not included. Chemists, pharmacists, medicine shops, private clinics that sold medicines, and agrovets that also carried human medicine were included. Public health facilities were interviewed after obtaining permission from the District Medical Officer of Health for Bungoma East district. The survey included elements from two questionnaires used in previous studies of retail outlets: (1) the ACTwatch Outlet Survey questionnaire [17] and (2) Provider Survey for the evaluation of subsidized ACT in retail outlets in Western Kenya [18].

All drug shops, pharmacists, chemists and private clinics are referred to as 'medicine retailers'. Those retailers that included the term 'clinic' in their name were relatively few and did not differ functionally from other retailers; they were no more likely to have diagnostics available or a trained health worker on staff. They sell medicines to their customers and are also a target for the AMFm subsidy so it seemed appropriate to analyze them with other retailers for the purpose of this study.

### Data analysis

Paper questionnaires were double entered into a Microsoft Access database and checked for consistency. Discrepancies were resolved by referring to the hard copies. Access tables were exported into Stata v10 for analysis. Maps were produced using ArcGIS10 (ESRI). The price of anti-malarials are reported in USD and KSh. The dollar value was arrived at using a conversion of 80 Ksh per USD.

### Ethical considerations

Prior to data collection, meetings were held with all the chiefs, assistant chiefs, and village elders in the communities where the study was proposed. Approval was sought from these community leaders and they disseminated information about the study to their communities. At the conclusion of the study, results were reported to the communities during health outreach activities. The study was reviewed and approved by Moi University Institutional Research and Ethics Committee and Duke University Institutional Review Board.

## Results

### Survey

117 medicine retailers were initially identified during the mapping. Of those, nine did not sell malaria medicines, 13 were found to have closed between the mapping (August) and the survey (November), and three were not found open after three visits. At the time of the survey, 11 new medicine retailers were identified that had been established between the mapping and the survey. There were 14 eligible public or mission facilities and 13 were available to be interviewed. In total, 130 staff members representing 97 shops and private clinics and 13 public/mission facilities were included in the analysis.

### Accessibility of facilities and medicine retailers

**Figure 1:**
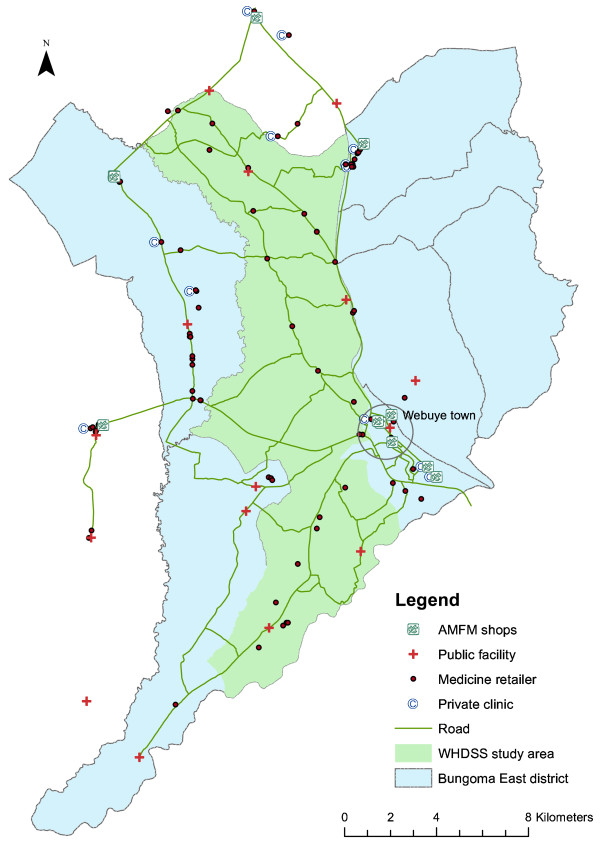
**Map of Webuye Health and Demographic Surveillance Site and surrounding district**. Public health facilities, private clinics, medicine retailers stocking AMFm AL, and all other medicine retailers are indicated.

Two dimensions of accessibility of public facilities and shops to the population they serve were measured - distance and operating hours. The Webuye DSS population is served by 13 government-owned health facilities; nine dispensaries, two health centres, one hospital and one sub-district hospital. There is one mission-run hospital in the northern part of the district. In addition to the public facilities, 97 medicine retailers were surveyed, including drug shops, pharmacies, chemists and private clinics (Figure 1). Most medicine retailers were located along the roads and near market centres. Based on the population estimates of the six sub-locations in the DSS area, there is approximately one health facility per 6,000 people and one shop per 800 people. Using the GPS coordinates of the facilities and medicine retailers and the GPS coordinates of the households in the WHDSS, the average distance from a household to a public health facility was estimated to be 2 km. The average distance to a medicine retailer was 1 km.

Most medicine retailers had weekend hours. 85% are open on Saturday and 42% are open on Sunday (including 6 of 12 private clinics). 85% were open after 5 pm on the weekdays and 8% were open after 7 pm on weekdays. Government-owned and mission facilities had more limited operating hours. Only public hospitals and sub-district hospitals were open on the weekend. Health centres and dispensaries open on weekdays between 8 am to 4 or 5 pm. One health centre and one dispensary reported having weekend hours.

### Availability of anti-malarials

For each retailer or facility, the types of medicines in stock on the day of the survey were recorded, stockouts of those drugs in the last one month, and drugs normally stocked but not available on the day of the survey (Table 1: shop characteristics). "Clinics" and all other retailers are described separately in the table to illustrate the similarity between these categories. However, since there is no functional difference between them as noted in the Methods section, they are grouped together for all other analyses.

**Table 1:** Shop and facility characteristics

	Shops	Private **clinics**^a^	Public facilities
**Total**	**85**	**12**	**13**

Open on Saturday	41	9	5
Open on Sunday	18	6	4
Open after 7 pm weekdays	4	0	0
Mean number of staff	1.4	2.2	3.2
Mean No. of anti-malarials in stock	2.8	2.4	2.8
Mean No. of anti-malarials out of stock	2.3	1.6	
AL in stock	37	6	13
SP in stock	48	7	7^b^
Artemisinin monotherapy in stock	4	1	0
Experience regular stockouts of all anti-malarials	40	3	5
Out of stock of all anti-malarials on day of survey	7	0	0
Mean price of AL	210 Ksh	175 Ksh	-^c^

a. Private clinic is defined as a retailer with the word 'clinic' in the name

b. SP is used for IPTp in public facilities

c. AL is free in public health facilities

On average, a malaria medicine retailer stocked three different types or brands of anti-malarials. SP was the most often stocked drug in the retail sector (32% of all malaria medicines available on the day of the survey), followed by quinine (30%). Forty-four percent of medicine retailers had artemether lumefantrine in stock that day, 57% had SP in stock, and 61% had quinine. 18 additional medicine retailers reported stocking AL but were out of stock on the day of the survey. Eleven retailers (11%) stocked the brand name "Artefan " which is the AMFm-subsidized brand of AL. These retailers were distributed throughout the study area (Figure 1). Other artemisinin combination therapies that were in stock at the time of the survey were dihydroartemisinin piperaquine (10 retailers), artemisinin piperaquine (one retailer), and artesunate - sulphamethoxypyrazine-pyrimethamine (one retailer). Only five malaria medicine retailers had uncombined artemisinin (5%). 3% of all drugs were past their expiry date. There were no medicine retailers with chloroquine.

Despite the fact that quinine was the second most frequently recorded drug and found in more retailers than other types of anti-malarials, only 12% of retailers reported quinine as the most commonly sold drug. 39% of malaria medicine retailers reported AL as the most frequently sold medicine and an equal percent reported SP as the most frequently sold anti-malarial.

Seven retailers (7%) normally stocked anti-malarials, but were completely out of anti-malarials on the day of the survey. Forty-four percent of malaria medicine retailers reported being stocked-out of all anti-malarials at some time, 14% reported this happening on a weekly basis and 25% reported this as a monthly occurrence. Five of 13 (38%) public health facilities reported being out of stock of all anti-malarials at some time. They reported this as either a weekly or monthly occurrence. For both retailers and government facilities, AL was not significantly more likely to have been out of stock in the past month or on the day of the survey than SP, amodiaquine or quinine.

When asked how they decide which drugs to stock, the overwhelming majority of respondents (65 of 97) reported customer demand as the driving factor, 27 reported being influenced by MOH guidelines or local health professionals, and eight reported wholesale price as being an important consideration. No respondents reported advertisement or incentives from pharmaceutical companies as influencing their decisions. The respondants were also asked how they decided which anti-malarial to dispense when multiple anti-malarials were in stock. Customer choice drove the decision about which anti-malarial to dispense for about half of respondents while 22 (18%) respondents reported weighing local health professional advice and 17 (13%) decided which anti-malarial based on the patient's symptoms.

### Affordability of anti-malarials

For each anti-malarial in stock, the price for a full treatment course was recorded. The mean price for a course of AL was 2.73 USD (SD = 1.95, Table 2). Prices for the subsidized brand of AL, Artefan, ranged from 0.5 USD to 2.5 USD (mean = 1.63 USD, SD = 1.3). For other artemisinin combinations, the mean price was 4.23 USD (SD = 0.84). The mean price for uncombined artemisinin was 5.40 USD (SD = 3.81). SP was the least expensive anti-malarial. The mean price for a dose of SP was 0.65 USD (SD = 0.33). Amodiaquine and quinine were slightly more expensive than SP, but still considerably less than AL (Table 2). Prices varied widely between shops for the same brandname and dosage form of a drug. A single brandname of quinine was found at 0.31 USD at one retailer and 5.63 USD in another. Similarly, a single brand of SP was between 1.13 USD to 3.75 USD and Coartem^®^, a brandname of AL, ranged between 0.63 USD to 7.5 USD.

**Table 2:** Prices of anti-malarials

	Mean price for treatment in USD (KSh)	Range USD (min, max)
**AL**	2.73 (218)	(0.38, 7.50)
*AMFm AL^a^*	1.63 (130)	(0.50, 3.75)
*Other*	2.86 (229)	(0.38, 7.50)

**ACT (non-AL)**	4.23 (338)	(3.13, 5.63)

**Artemisinin** **Monotherapy**	5.40 (432)	(1.50, 10.5)

**SP**	0.65 (52)	(0.19, 1.50)

**Quinine**	1.21 (97)	(0.25, 5.63)

**Amodiaquine**	0.80 (64)	(0.38, 1.13)

*^a^*Artefan, AMFm subsidized brand

If a client didn't have enough money for the medicine, 63% of retailers said they would give on credit and 18% of shop workers said they would offer a cheaper alternative or sell them only the number of tabs they could afford. 28% said they have sold a smaller dose when a customer demanded it and 57% said they have separated tabs from packaging to sell to customers. Only 13% of respondents said they would refuse to sell at all if the customer could not afford the medicine. 61% of respondents said they would refer a customer to another shop or a health facility if they were out of stock of all anti-malarial medicines.

## Discussion

This study explored three dimensions of access to anti-malarials through the retail sector in a rural district in Kenya following the implementation of a nation-wide anti-malarial subsidy programme. Accessibility, availability and affordability anti-malarials in the retail sector was compared to the public sector facilities in the same district.

In this study, 'accessibility' is defined as physical access to an outlet where anti-malarials may be obtained. Alba *et al *reported that physical access was the most important factor in determining whether a patient got an effective anti-malarial. Patients living in a village with a shop or health facility were four times more likely to get an ACT within 48 hours of onset of fever [19]. Based on the results presented here, anti-malarials may be more accessible through the retail sector when considering the abundance of malaria medicine retailers, their proximity to households, and longer opening hours than public facilities. On average, households travel twice the distance to attend a public health facility than to reach a malaria medicine retailer. Most public health facilities do not offer weekend opening hours. Hospitals and health centres do open on Saturdays and Sundays (hospitals only), but these facilities are few and located near the peri-urban centre, away from the rural communities. In contrast, 85% of the malaria medicine retailers offer Saturday opening hours and nearly half open on Sunday, a factor shown to contribute to treatment seeking in the retail sector in other studies [2].

AL has been the recommended first line treatment for uncomplicated malaria in Kenya since 2006. SP is still widely available and used, but resistance is common; more than 80% of infections are resistant [20] resulting in 22-40% treatment failure [21,22]. Forty-four percent of malaria medicine retailers had AL in stock on the day of the survey, and an additional 20% reported stocking it but were out of stock that day. Thirty-nine percent of malaria medicine retailers reported that AL was the most commonly sold drug. In contrast, a study in a neighboring district showed almost no retail sales of AL in 2008, only two years before our study [18].

Only seven malaria medicine retailers were completely stocked out of anti-malarials on the day of the survey, although a quarter reported stockouts as a regular occurrence. SP and quinine were the most frequently stocked drug, together making up over 60% of all the drugs observed. On a positive note, very few malaria medicine retailers sold uncombined artemisinin and none reported carrying chloroquine.

In public facilities, AL, SP, and quinine injections were available. SP is provided to facilities for Intermittent Preventive Treatment in pregnancy (IPT-p) although it cannot be ruled out that SP is being given for febrile episodes. Although only five out of 13 public facilities reported regular stockouts of anti-malarials, anti-malarial shortages are a frequent problem in rural facilities in Kenya [23].

Prices of anti-malarials varied widely between types and between retailers. The same brandname sometimes varied by an order of magnitude between retailers. It is possible that medicine retailers procured the same brandname from different wholesalers or other outlets leading to price variability. It is likely that prices in the retail sector are not 'fixed' at the level of the consumer, particularly in the less formal retail shops, but rather the price may be adjusted based on the client's ability to pay. Other studies have shown that the decision about which drug to sell is often dictated by what the patient can afford. In our study, medicine retailers reported separating blister packs of pills and selling only the number of tablets that a client can afford. Overall, less effective anti-malarials like SP and amodiaquine were significantly less expensive than artemisinin combination therapies. Non-AL ACT was more expensive and less common. Uncombined artemisinin was more expensive than AL or other types of ACT. In contrast to our results, a study in Burundi found ACT in private retail shops to be less expensive than quinine and amodiaquine even without subsidies [14]. In Kenya, the minimum wage is 2.5 USD per day. Although the average price of AL is roughly equivalent to one-day's wages, a criteria for affordability proposed by the World Health Organization, unemployment in Kenya is estimated to be 40% and most casual workers found in our study area do not work every day of the month. In the WHDSS area, the high price of ACT is undoubtedly still an obstacle to accessing effective treatment.

Pharmacies in Kenya are required to be registered and only registered pharmacies are permitted to sell prescription medicines. Even registered pharmacies in Kenya are not legally permitted to sell ACT over-the-counter without a prescription and unregistered medicine retailers are not permitted to sell ACT at all, although both practices are common. Under the current policy, the AMFm subsidy would only benefit patients who have a prescription either from a private facility or from a public facility where AL was unavailable. There is some misalignment between current policy in Kenya and the intention of the AMFm subsidy. Tanzania has designated a special cadre of accredited shops that are permitted to sell ACT over the counter. Establishment of Accredited Drug Dispensing Outlets (ADDOs) and provision of subsidized ACT through this channel has greatly improved and expanded access to ACT [8,19]. However, the strategy hasn't significantly reduced the percent of older children and adults who purchase SP for their fever [19] and only 30% of drug shops carried AL [24]. The survey used for this report did not specifically identify registered pharmacies, but according to the Kenya Pharmacy and Poisons Board there are only six pharmacies registered in the area, indicating that the large majority of medicine shops in our study were probably unregistered. Formalizing the sale of ACT over-the-counter in Kenya through specialized or trained drug vendors may increase the accessibility and availability of subsidized AL.

Providing subsidized ACT through the retail sector is intended to reduce the cost of effective drugs to below that of ineffective therapies and increase the number of fevers treated with an appropriate anti-malarial. A randomized controlled trial in Kenya demonstrated a dramatic improvement in the percent of children under-5 who received AL for their fever after deployment of subsidized AL in the retail sector [18]. A pilot evaluation of subsidized ACT provided to wholesalers in three districts in Tanzania also showed dramatic improvements in the number of patients who purchased ACT [25].

This is the first report of the effect of the international subsidy of ACT through the retail sector. Country-wide retail sector subsidies for AL through the AMFm programme began in Kenya in August 2010, five months before our survey. Between August and November 2010, 1.2 million treatment courses of subsidized AL (a single brandname - Artefan) were procured and delivered to Kenya. National media campaigns were used to raise public awareness about the subsidy and the correct pricing. The price of a treatment course of AL under the subsidy is intended to be 0.5 USD (40 Ksh). In this study, subsidized AL was significantly less expensive than other brands, but still almost three times as expensive as SP. However, the subsidy is applied at the level of the wholesaler, and retailers set their own prices. In our survey, only one shop was selling subsidized AL at the recommended price.

Eleven percent of malaria medicine retailers in the study area stocked subsidized AL. Nearly all retailers with subsidized AL did not carry non-subsidized brands (either in-stock or reported out-of-stock) which suggests that these retailers may not have been selling AL prior to the subsidy and the subsidy may have expanded the availability of AL. In addition, retailers with subsidized AL were distributed throughout the study area (Figure 1) and were not concentrated in the town centre.

It is interesting to speculate about how accessibility, availability, and affordability impact treatment-seeking decisions for febrile illnesses. Clearly, accessing anti-malarials through medicine retailers is more convenient than attending a public health facility for most families. On the other hand, treatment is free in public facilities, although families must also weigh transportation costs and time investment to attend the public facility [2]. It is also possible that patients perceive 'free drugs' as less desirable or ineffective [26,27]. During frequent drug shortages in public facilities, patients receive a prescription and must buy the drug from a retailer. When drugs are not available in facilities, many patients probably bypass the facility and go directly to the retailer.

Our results also demonstrate an impact in the reverse direction - customer demand, preference, and resources influence which drugs are stocked in retail shops and which drug a customer purchases. This is in agreement with an exit survey of shop customers, which showed that the majority of patients who visited a shop specifically asked for an anti-malarial, but only 16% asked for an ACT. Asking for an ACT significantly increased the likelihood of receiving one [14]. Shops are responsive to customer demands and preferences which suggests that leveraging customer awareness and demand could have a significant positive impact on the effectiveness of the AMFm subsidy. This also underscores the importance of continuing public awareness campaigns and health education messages in the implementation of AMFm.

## Conclusions

In Kenya, only 12 percent of children receive an appropriate anti-malarial for their fever within 48 hours of onset [28]. The retail sector is an important source of anti-malarials for fevers. Treatment was sought from a local medicine retailer for 45% of children with fever in a nearby rural district in Kenya [5]. A review of treatment-seeking behaviour for fevers in Kenya reported that between 17 and 83% of fevers are treated with medicines purchased from shops [3], including anti-malarials. Improving access to effective anti-malarials through the retail sector could be an efficient way to increase the percent of fevers that are treated promptly and effectively.

The results reported here describing access to anti-malarials in the retail sector generally agrees with previous work. Anti-malarials in the retail sector are more accessible but less affordable than the public health sector. First-line therapy for uncomplicated malaria, AL, is available in nearly half of malaria medicine retailers, but is still more expensive than ineffective drugs. Encouragingly, subsidized drugs had already penetrated the market even in this rural community only five months after the launch of the subsidy. There is an opportunity to improve the impact of the subsidy by leveraging customer demand to increase the number of retailers stocking AL.

## Competing interests

The authors declare that they have no competing interests.

## Authors' contributions

NS participated in study design, led development of the data collection tool, collected the data, and provided critical revisions to the manuscript. AO participated in developing the data collection tool, drafting the manuscript, and interpreting the results. CS participated in data collection and interpreting of the results. DM participated in developing the data collection tool and interpreting results. BO participated in developing the data collection tool and interpreting results. WPO conceived the study, participated in study design and developing the data collection tool, performed the analysis and drafted the first version of the manuscript. All authors read and approved the final manuscript.
